# Unlocking asylum seekers’ voices: protocol of a mixed-method clinical study on the use of the cultural formulation interview with asylum seekers in Belgium

**DOI:** 10.3389/fpsyt.2023.1156803

**Published:** 2023-05-04

**Authors:** Lukas Claus, Meryam Schouler-Ocak, Mario H. Braakman, Bernard Sabbe, Godfried Van Beuren, Seline van den Ameele

**Affiliations:** ^1^Collaborative Antwerp Psychiatric Research Institute (CAPRI), Universiteit Antwerpen (UA), Antwerp, Belgium; ^2^Department of Psychiatry, Vrije Universiteit Brussel (VUB), Universitair Ziekenhuis Brussel (UZ Brussel), Brussels, Belgium; ^3^Department of Psychiatry and Psychotherapy, Psychiatric University Clinic of Charité at St. Hedwig Hospital, Berlin, Germany; ^4^Department of Psychiatric Residency Training, Pro Persona Mental Health, Wolfheze, Netherlands; ^5^Department of Criminal Law, Tilburg Law School, Tilburg University, Tilburg, Netherlands; ^6^Management Culturally Sensitive Care, St. Alexius Psychiatric Hospital, Grimbergen, Belgium

**Keywords:** cultural formulation, cultural formulation interview, mental health, cultural psychiatry, psychiatric diagnosis, asylum seekers

## Abstract

**Background:**

Despite a high prevalence of mental disorders among asylum seekers, many barriers to mental healthcare exist. Cultural and contextual factors strongly influence the experience and expression of psychological distress, putting asylum seekers at greater risk of misdiagnosis and inappropriate treatment. The Cultural Formulation Interview (CFI) is a useful tool to map out cultural and contextual factors of mental disorders; however, to the best of our knowledge, it has not yet been investigated in asylum seekers specifically. The primary aim of this study is to evaluate the value of the CFI in the psychiatric assessment of asylum seekers. Second, we will describe the themes relevant to psychiatric distress in asylum seekers that are identified by the CFI. In addition, asylum seekers’ experience of the CFI will be evaluated.

**Methods and analysis:**

This cross-sectional, mixed-method clinical study aims to recruit a group of 60–80 asylum seekers (age 15–29) with mental health symptoms. Data will be collected using structured (MINI, PCL-5, HDRS-17, WHOQoL-BREF & BSI) and semi-structured (CFI & CFI-debriefing) questionnaires to assess cultural background, contextual factors, and illness severity. Multidisciplinary case discussions will be held after the completion of interviews, following a methodological stepped approach. Combining qualitative and quantitative research techniques, this study aims to generate reliable knowledge on working with the CFI in asylum seekers. Based on the findings, recommendations for clinicians will be developed.

**Discussion:**

This study addresses the knowledge gap on using the CFI in asylum seekers. Compared to prior studies, it will provide new insights into the use of the CFI in the specific context of working with asylum seekers.

**Ethics and dissemination:**

Prior research on the CFI in asylum seekers is limited, partly because of their high vulnerability and low access to care. The study protocol has been tailored in close collaboration with several stakeholders and validated after piloting. Ethical approval has already been obtained. Together with the stakeholders, the results will be translated into guidelines and training materials. Recommendations to policymakers will also be provided.

## 1. Introduction

Over 880,000 people applied for international protection across Europe in 2022, with more than 35,000 of those applications being made in Belgium. These are the highest number since the 2015 refugee crisis (1, 2). Asylum seekers are exposed to numerous risk factors for psychopathology, such as trauma, lack of shelter, uncertainty, and the long duration of the asylum procedure (3). Psychopathology is highly prevalent among asylum seekers, with prevalence rates up to 30% for post-traumatic stress disorder (PTSD) and depressive disorder (4). However, their use of mental health services is low compared to the need (5, 6). The underutilization of mental health services may be explained by specific barriers, such as lack of knowledge of the healthcare system, language barriers, lack of trust toward authority, structural difficulties (financial limitations, precarity, and lack of service capacity), and discrepant beliefs and expectations of mental health and healthcare (7, 8).

It can be hard for care providers to distinguish between psychiatric disorders and symptoms of temporary distress (9), or to recognize certain manifestations of psychological distress, such as somatization (8). Culturally compliant reactions can be wrongly assessed as pathological (10, 11). Asylum seekers may rely on other explanatory models, e.g., of a religious or supernatural nature (8, 10). A culturally determined different notion of mental health can lead to a feeling of incongruence between the care system and the experienced needs (5, 12, 13).

A vast number of studies demonstrate that immigrants and ethnic minority patients are at higher risk of being misdiagnosed, in particular refugees and recently arrived immigrants (14–16). Classical DSM diagnosis, based on decontextualized criteria, may fall into a category fallacy and fail to characterize an individual’s experience (17). More attention to the impact of culture and context can be helpful to improve diagnostic assessment and treatment planning (18). The Cultural Formulation Interview (CFI) is a valuable instrument for this purpose (19).

The DSM-IV Outline for Cultural Formulation (OCF) provided a framework for assessing the cultural features of an individual’s mental health problem and how it relates to its social and cultural context and history. To operationalize the OCF for clinical practice, it was revised into the DSM-5 CFI (20). This questionnaire focuses on the patient’s perspective and social context during psychiatric evaluation and allows patients to narratively describe their experiences (21). Although the effects of CFI implementation on health services and clinical outcomes have been scrutinized, there is still a need to document the specific clinical advantages it offers (22).

The CFI was evaluated among diverse migrant populations, revealing themes such as cultural identity, trust, stigma, and psychosocial needs to be important for improving understanding and therapeutic relationships (23, 24). The DSM-5 field trial found the CFI to be feasible, acceptable, and useful (21). According to a Swedish study, the CFI effectively makes psychiatric assessments more patient-centered by facilitating patients’ illness narratives (25). However, other studies also identified barriers, such as a mismatch between the CFI questions and the understanding of culture held by Mexican clinicians or a lack of suitability for severely ill, psychotic patients (20, 26). Patients appreciated the CFI’s recognition of their cultural identities and illness narratives and an approach of curiosity and empowerment guided by the CFI, which made them feel dignified, hopeful, and engaged in future care (27).

To date, the evidence of the CFI’s impact on clinical outcomes remains limited (22). Two studies report improved patient-clinician communication (28, 29). Different case reports describe how the CFI can change clinical service delivery and impact diagnostic formulation and treatment adherence (22, 24, 30, 31). Two studies revisited diagnostic categorization after using the older DSM-IV-TR Cultural Formulation, leading to a revision in about half of the cases (14, 15). To date, only one study evaluated whether the DSM-5 CFI impacted the diagnostic process. They found that the CFI may help identify depressive disorder in non-native-speaking patients in a migration context (32). Lindberg et al. confirm that the CFI approach is particularly important in vulnerable and asymmetrical encounters with migrants or other marginalized groups (27). There remains a need to document its distinct clinical benefits and to study how the CFI can be used in diverse settings with specific subpopulations (22).

In this study, we aim (1) to determine the value of the CFI in the diagnostic assessment of asylum seekers with psychiatric symptoms (main research aim), (2) to identify the main themes that emerged from the CFI with asylum seekers, and (3) to evaluate asylum seekers’ experience of the CFI. We hypothesize that the CFI is an acceptable and feasible instrument to enhance sensitivity to cultural and contextual factors in mental healthcare for asylum seekers and impacts the diagnostic process. However, its use among asylum seekers has at the best of our knowledge not been studied until now and its value in diagnostic assessment is still unclear.

## 2. Methods and analysis

### 2.1. Overview of the study design

In a cross-sectional clinical study design, we will recruit a group of 60–80 asylum seekers with mental health symptoms. Using a mixed-method approach with structured, semi-structured, and open questionnaires we will assess the cultural background, contextual factors (e.g., illness explanation, identity, and migration history), mental health symptoms, and illness severity. After conducting the interviews with each participant, the results will be discussed during a multidisciplinary case discussion.

A descriptive approach will map out the main themes related to psychiatric distress in the illness narratives of asylum seekers that are identified by the CFI. An evaluation study will assess the experience of the CFI in asylum seekers using both quantitative (acceptability, feasibility, and utility) and qualitative methods, and comparing results among any relevant subgroups (e.g., according to illness severity). Based on the reports of the multidisciplinary case discussions, we will analyze the impact of the cultural and contextual information obtained through the CFI on the diagnostic assessment. To ensure the implementation of the results, we will organize a participative trajectory with field workers to translate our findings into recommendations.

### 2.2. Study context

This study, set in routine clinical care for asylum seekers, differs from prior research on the CFI that was majorly limited to academic settings (22). On one side, stakeholders from the asylum reception sector in Belgium (Fedasil, Red Cross Belgium and Caritas Belgium) were approached. On the other side, the project is strongly embedded in an initiative that provides psychiatric care for asylum seekers in Belgium (POZAH project, Psychiatric Hospital Sint-Alexius Grimbergen). Based on the experiences and needs of these stakeholders, the research proposal was developed. Following the development of the study, the research proposal has been discussed with the (inter) national partners and adjusted to ensure the feasibility and relevance of the research project.

### 2.3. Study procedure

#### 2.3.1. Study population and consent procedure

Inclusion and exclusion criteria are described in Table 1. First-line healthcare workers and social workers of the asylum centers refer asylum seekers to the research project in case they estimate that a psychiatric assessment is indicated because of suspected severe mental distress. Since all patients referred to the POZAH project meet this condition, participation is proposed to all of them in case they meet the inclusion criteria. By involving both the asylum centers and a clinical setting, we aim to recruit a clinically representative sample of asylum seekers with mental health symptoms. Following referral, the research team will determine whether a participant meets the in-and exclusion criteria listed in Table 1. This study will focus on adolescent asylum seekers and unaccompanied minor refugees (15–29 years old), as evidence suggests that this group is at high risk for developing serious and long-term mental health issues (33). An age criterion was also set forth by the funder of this study.

**Table 1:** Inclusion and exclusion criteria.

Inclusion criteria	Exclusion criteria
The participant is between 15 and 29 years old	The participant is experiencing acute suicidality
The participant meets criteria of a psychiatric condition^*^	The participant is clinically intoxicated
The participant is in an asylum procedure in Belgium	The participant is in withdrawal of substances OR
The participant is able to give written informed consent AND	The assessment is impossible due to cognitive deficits (severe cognitive disability)
The participant is capable of oral communication with the researcher in Dutch, English or French, or with an interpreter.	

^*^The need for psychiatric referral as estimated by the involved caregiver in the asylum center or hospitalization in a psychiatric ward.

If willing to participate after a time of consideration, patients once more receive verbal and written information about this study during their first appointment with the interviewer. The written information is available in Dutch, French, English, Dari, Pashtu, and Arabic (classic). Translations have been performed by certified translators and double-checked by native speakers. During the first appointment, special attention is paid to ensure that the information on the study is easily understandable and culturally sensitive. Consequently, they are asked for their informed consent. Written informed consent implies voluntary participation, the freedom to withdraw at any time, and the absence of treatment or procedural implications for non-participation. Contact information is provided to caregivers and participants to allow them to request additional information or contact the researchers.

Talking about sensitive issues may trigger difficult emotions and traumatic memories, which could increase the mental burden of the participant. Therefore, the interview can be paused, or the participation interrupted if needed. To monitor this appropriately, reasons for drop-out will be carefully documented.

#### 2.3.2. Clinical assessment

**Figure 1:**
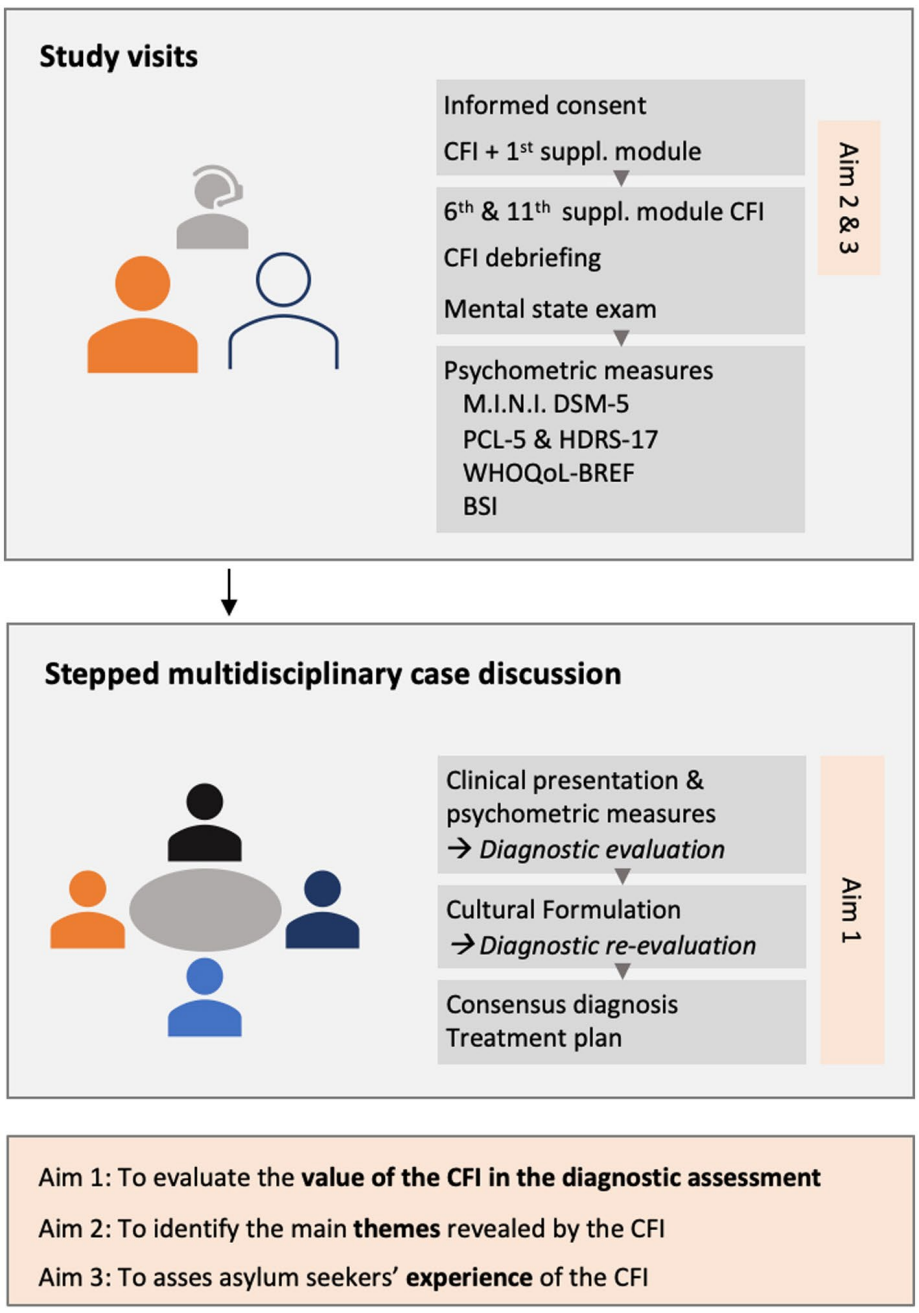
Overview of the study design, interviews, multidisciplinary case discussions, and research aims. CFI, DSM-5 Cultural Formulation Interview; M.I.N.I. DSM-5, Mini International Neuropsychiatric Interview for DSM-5; PCL-5, PTSD Checklist for DSM-5; HDRS-17, 17-item Hamilton Depression Rating Scale; WHOQoL-BREF, World Health Organization Quality of Life Questionnaire-BREF; BSI, Brief Symptom Inventory.

Following the information and consent procedure, a researcher-psychiatrist will conduct a clinical assessment. This assessment is split into 3 interviews, each with an estimated duration of 1.5 h (see Figure 1). Certified interpreters are used if the participant is not fluent in Dutch, French, or English. Participants will receive an incentive of 15 euros per interview completed.

First, the researcher-psychiatrist will administer the DSM-5 CFI, completed by the first (explanatory models) and sixth (cultural identity) supplementary module.

The second session will start with the administration of the 11th (migrants and refugees) supplementary module. Subsequently, the participant’s experience of the CFI will be debriefed using a set of open and closed questions. A mental state examination will also be taken.

The last session consists of a standard structured psychiatric assessment. The instruments are described below.

The informed consent explicitly mentions that following the interview the main issues will be discussed with the referring caregivers of the participant to ensure adequate follow-up.

#### 2.3.3. Multidisciplinary case discussions

Formulating a diagnosis in mental health is an act of double interpretation, as it relies on the clinician’s interpretation of the patient’s understanding of his difficulties (34). To answer the main research question on the value of the CFI in the diagnostic assessment, we aim to evaluate the process of clinical interpretation. The limited literature proposes multidisciplinary case discussions as a possible approach to this challenge (35). Multidisciplinary case discussions on the CFI may help to promote a more complex and nuanced vision and contribute to diagnostic shifts. These discussions can help to avoid a “culturalizing” bias, which would attribute adaptation difficulties solely to cultural differences. Instead, multidisciplinary case discussions may support the framing of difficulties in a broader social and cultural context (35). Multidisciplinary case discussions also permit the objectification of the reasons for diagnostic revision (15).

Building on the existing evidence, we developed a methodological stepped approach (see Figure 1). The interviewer will conduct structured clinical case discussions together with the referring care provider, a psychiatrist with expertise in cultural psychiatry, and an independent psychiatrist. The discussion will start with the reason for referral given by the care provider, the presentation of the main complaints, a mental state examination, the MINI diagnoses, and the results of the symptom severity scales. Based on this clinical information, the panel will formulate a clinical diagnosis (Step 1). Next, the interviewer will present the content of the CFI, after which the panel will discuss the cultural formulation, the need and reasons for revision (Step 2). The panel will then conclude with a consensus on a final diagnosis, which will be compared to the initial diagnosis (Step 3).

#### 2.3.4. Participative trajectory

During workshops with caregivers from participating asylum centers and hospitals, the findings will be translated into practical guidelines. Both the results of the content and the experience of the CFI will be discussed. The goal is to optimize mental healthcare for asylum seekers by discussing the most pressing needs and addressing barriers to CFI implementation. Identification of needs and barriers will enable the formulation of recommendations for different policy levels.

### 2.4. Sample size

From a quantitative perspective, the main research question involves the prevalence of diagnostic revision. Previous research based on the DSM-IV OCF suggests that about 50% of the participants underwent diagnostic revision, although the prevalence of diagnostic revision after DSM-5 CFI administration seems more limited (14, 15, 32). Based on a supposed effect size of 0.25, sample size calculation based on a one sample t-test for means with SPSS learns that a sample of at least 73 individuals is needed to estimate the true frequency of revision with a precision of 10% (assuming a confidence level of 95%).

From a qualitative point of view, it is important to ensure that the sample is adequately diverse. For obtaining this diversity, we aim to include a group of 20 non-Afghan asylum seekers. In 2021 20% of adult asylum seekers and 75% of non-accompanied minor refugees had the Afghan nationality (36). Supposing half of the included participants will be minor, a total number of 60 participants should largely suffice to have included minimum 20 non-Afghan asylum seekers. This sample size should guarantee adequate stretch of our hypotheses and achievement of thematic saturation (37), which will be monitored during the study (38–40). Furthermore, sufficient diversity allows for comparison between specific subgroups.

Considering the feasibility and limits (time, funding) of this study, the goal is to include a minimum of 60 and maximum of 80 participants over 18 months. A pitfall in obtaining the needed number of inclusions is the dependence on our partners. If insufficient inclusions result, recruitment sites will be expanded. The stigma that sticks to mental healthcare can also be a barrier to inclusion. For this reason, we will hold on to our decision to recruit participants through referral by trusted caregivers.

### 2.5. Applied instruments

#### 2.5.1. DSM-5 cultural formulation interview

The cultural formulation interview (CFI) is a standardized 16-item questionnaire designed to focus attention on the patient’s perspective and social context during psychiatric assessment in a narrative way (41). The questionnaire covers 4 cultural domains: (a) definition of the problem; (b) perceptions of cause, context, and support; (c) factors affecting self-coping and past help-seeking; and (d) factors affecting current help-seeking (21). As it has been designed as an intake instrument, the assessment in this study will start with the core CFI (42).

The CFI has 12 supplementary modules addressing specific topics or populations, depending on what areas of an individual’s problems clinicians want to elaborate. To answer our main research question comprehensively, it was decided *a priori* to administer the first (explanatory models) and sixth (cultural identity) supplementary module. The 11th module (immigrants and refugees) will be used as a proper assessment of the migration trajectory (43).

The first supplementary module aims to clarify the individual’s understanding of the problem based on his or her ideas about cause and mechanism (explanatory models) and past experiences (illness prototypes). The sixth module aims to understand an individual’s cultural identity and how it has affected their health and well-being. The 11th supplementary module intends to gain insight into pre-migration difficulties, migration-related losses, and challenges, in the ongoing relationship with the country of origin, the resettlement, the relationship with the mental problem, and the future expectations (43). Validated translations of the core CFI and supplementary modules are available in Dutch, French, and English.

#### 2.5.2. CFI debriefing

For the evaluation of the experiences of the CFI by asylum seekers, we will combine 2 instruments. The first instrument is the debriefing instrument for patients (DIP), which has been developed in the DSM-5 field trial (21) and was adapted by Wallin et al. to improve its relevance and comprehensibility (25). This version will be used to quantify acceptability, feasibility, and utility. The second instrument is the open CFI-debriefing interview focusing on the perceived importance of content, emotions, cognitions, utility, and distinctiveness, as proposed by Muralidharan et al. (31).

#### 2.5.3. Psychometric scales

##### 2.5.3.1. M.I.N.I. 7.0.2 DSM-5

The Mini-International Neuropsychiatric Interview (M.I.N.I.) is a short structured diagnostic interview for DSM-5 psychiatric disorders. It was designed to meet the need for a short but accurate structured psychiatric interview for clinical trials and epidemiology studies (44, 45). The interview’s brevity makes it especially suitable for diagnosing psychiatric patients in everyday clinical practice (46). In recent years, the assessment tool is also being used in humanitarian aid and global health settings (47). Validation and reliability studies have been done to compare the MINI to the SCID (Structured Clinical Interview for DSM). The results of these studies show that the MINI has similar reliability and validity but can be administered in a much shorter period (44). Validated translations of the MINI (English, French, and Dutch) will be used (46).

##### 2.5.3.2. PTSD checklist for DSM-5

The PTSD Checklist for DSM-5 (PCL-5) is a standardized assessment tool to assess the presence and severity of PTSD-symptoms. It consists of 20 items that evaluate the five PTSD-symptom clusters. The PCL-5 has been shown to effectively identify PTSD in individuals from diverse cultural backgrounds and has different validated translations (48, 49). The total score ranges from 0 to 80. A cut-off score of 33 determines the presence of PTSD. Although there are no established severity ranges for the PCL-5, a higher score indicates higher symptom severity (50).

##### 2.5.3.3. Hamilton depression rating scale 17-items

The Hamilton Depression Rating Scale with 17 items (HDRS-17) (51) will be used to determine the severity of depressive symptoms. Although the core questions on depressive disorder (depressed mood, guilt, loss of interest, and retardation) appear pertinent to the assessment of depression across cultures, the psychometric properties of the full HDRS-17 are still debated (52). Since HDRS-17 is frequently used in routine clinical care, the administration of this questionnaire has been chosen to allow describing the differences from the current standard of care. Recommended severity range for the HDRS-17 is no depression (0–7), mild depression (8–16), moderate depression (17–23), and severe depression (≥24) (53).

##### 2.5.3.4. WHOQoL-BREF

The WHOQoL-BREF questionnaire is a shortened version of the World Health Organization Quality of Life (WHOQOL) assessment (54). It consists of 26 items that assess an individual’s perception of their overall quality of life and well-being across four domains: physical health, psychological health, social relationships, and environment. This information can be used to identify areas of strength and areas in need of support. The WHOQOL-BREF questionnaire has been translated into over 50 languages and has been used in a wide variety of cultural contexts (55).

##### 2.5.3.5. Brief symptom inventory

The Brief Symptom Inventory (BSI) is a standardized tool that consists of 53 items measuring symptom severity on 9 symptom dimensions (56). The BSI can be used to calculate the Global Severity Index, which is an indicative measure of general symptom severity. Validated translations (English, French, and Dutch) will be used (57).

### 2.6. Data collection and management

The interviews will be recorded and transcribed, after which the original audio will be deleted. Data will be managed using REDCap, an electronic data capture tool hosted by the University of Antwerp. REDCap is a secure, web-based platform that supports data capture for research studies (58). The tool logs the history of data entry and offers automated procedures for exporting data to statistical software. NVivo will be used for the analysis of qualitative data (59), and SPSS for quantitative data (60). Digital research data will be stored for a period of 20 years at an encrypted cloud service provided by the University of Antwerp, while paper informed consent forms will be kept under lock and key for a period of 5 years.

### 2.7. Data analysis

#### 2.7.1. Qualitative

The approach of the CFI emphasizes intersubjectivity and the inherent difference between the clinician and the patient (alterity) (61). This approach calls for efforts to jointly explore and co-construct meaningful narratives (62). It emphasizes the embedding of a diagnosis in a particular time, context, and culture (63). As this project is based on this construction of meaning and knowledge through the interaction between the participant, the clinicians, and the researcher-psychiatrist, it generally takes an epistemological social constructivist stance, considering the knowledge to be negotiated between people and within a given context and time frame (64).

##### 2.7.1.1. Question 1: a thematic analysis of the CFI

A thematic analysis will be conducted to identify themes related to psychiatric distress that are added by the CFI to the illness narratives of asylum seekers (65, 66). Braun and Clarke’s six-phase approach to thematic analysis (Braun and Clarke, 2006) will be applied. The steps include familiarization with the data, creating initial codes, identifying and reviewing themes, naming and explaining each theme, and writing a report. A central organizing concept will be defined to explain and connect the supporting quotations within each theme (66). Braun and Clarke describe coding as an active and reflexive process that reflects the researcher’s interpretations of patterns of meaning across the dataset (67). Codes will be compared and discussed until a consensus is reached. To ensure the rigor of the analysis, we will have discussions among the researchers to address any issues with the coding or themes. The results of the analysis will consist of higher-level themes and categories that will identify themes added by the CFI to the illness narrative of asylum seekers. During the analysis process, existing models of thematic concordance and data quality will be used to ensure that the sample size is sufficient, the data are adequate, and the themes are saturated (38–40, 68). The intent is to collect sufficient data to have common themes present across the entire dataset, without any new themes being produced by analyzing the last transcript.

##### 2.7.1.2. Question 2: asylum seekers’ experience of the CFI

The qualitative data analysis relates to the data from the semi-structured, open CFI debriefing interviews. The quantitative analysis of acceptability, feasibility, and utility is described below. A thematic framework analysis will be used to map out the perceptions of the CFI administration and the influencing factors on the perception of asylum seekers (69). The framework method is not tied to any specific epistemological or philosophical approach. It is a flexible tool to facilitate constant comparative techniques by reviewing data across a matrix (70). It is particularly useful for evaluating a service or getting structured answers to research questions, like identifying barriers and facilitators. Comparing and contrasting data is a key part of qualitative analysis, and the framework method is designed to facilitate this process across and within cases (70). This method involves five stages for coding and analyzing the data: familiarization, identification of a thematic framework, indexing, charting, and mapping and interpretation (69).

Taking into account a possibly relatively small amount of data per interview question due to their structured nature, a larger number of participants (30 to 60) may be needed to achieve saturation (40). Data will be organized in charts and analyzed using matrices to identify patterns. Illustrative quotations will be included to represent the themes and analysis. Comparison will pay particular attention to the role of participants’ background and illness severity in their experience of the CFI.

##### 2.7.1.3. Question 3: the value of the CFI in the diagnostic assessment of asylum seekers with psychiatric symptoms (main research question)

The evaluation of the value of the CFI in the diagnostic assessment will be based on the reporting of the multidisciplinary case discussions. These reports consist of our stepped approach (Figure 1): (1) a description of the clinical picture and tentative diagnosis, followed by (2) a CFI-based cultural formulation of the symptoms, and (3) a re-evaluation of the diagnosis based on this case formulation. To enable a description of the role of the CFI, these reports will undergo qualitative content analysis. Qualitative content analysis is a research method for the subjective interpretation of the content of text data through a systematic process of coding and identifying recurring patterns or themes (71). For this study, conventional qualitative content analysis will be used to describe a phenomenon, in this case, the CFI’s value in the diagnostic process (71). Conventional content analysis is usually appropriate when an existing theory or research literature on a phenomenon is limited. It allows the categories and names for categories to be produced from the data, which has also been described as inductive category development (72). Conventional content analysis allows the researcher to gain a more in-depth understanding of a phenomenon (71).

Mayring has developed a model of qualitative content analysis that includes three distinct procedures: (a) summary, which involves reducing and paraphrasing the material; (b) explanation and clarification of the material; and (c) structuring, which involves filtering out a particular structure from the material and examining it (73). During structuring, codes will be assigned to segments of text to identify patterns and themes. The codes will be grouped into clusters, providing a broad overview of the data. The codes and clusters can be arranged to show the relationships between them, but the main goal is to determine the frequencies of the codes rather than the specific meaning behind (71).

Considering the high degree of diversity of our sample, which guarantees the maximal stretch of the hypotheses, it is necessary to aim for a big enough sample. Insufficient saturation can lead to analytic difficulties in conventional content analysis (74). As for question one, existing models (38–40) will be used to ensure themes are saturated. Qualitative content analysis of 60 to 80 participant records seems a feasible goal that can be expected *a priori* to suffice for obtaining thematic saturation.

#### 2.7.2. Quantitative data analysis

Quantitative data consist of scores on the debriefing instrument for patients (DIP), on the psychometric scales, and of the presence of diagnostic revision. To allow a better understanding of our study sample, an introductory descriptive analysis will characterize the background of the participants, such as their age, gender, education level, country of origin, and procedural status. The diagnoses and symptom severity of the study sample, as well as other relevant variables like treatment history or the use of psychopharmaceutical drugs, will be outlined.

For the CFI debriefing instrument for patients, the negatively worded item ‘Took more time to share my perspective than I wanted’ will be scored in reverse. Means and standard deviations for each item and for the factors of Clinical Utility and Feasibility will be computed. Where applicable, the internal consistency of the items will be assessed using Cronbach’s alpha. Based on characteristics such as illness severity (based on scores on PCL-5, HDRS-17, and BSI), psychotic vs. non-psychotic disorder, and minor vs. adult, subgroups will be determined and differences in the experience of the CFI between these subgroups will be evaluated.

The presence of diagnostic revision is determined by the difference between the clinical and the culturally sensitive diagnosis as discussed during the “stepped” multidisciplinary case discussion. It can be expected that a case has a principal diagnosis and one or more differential diagnoses at both moments of the assessment. For this study, a change of diagnosis will be determined as a change in DSM-5 diagnostic category (e.g., trauma-and stressor-related disorder, depressive disorder, etc.). Post-hoc comparisons of sociodemographic and clinical variables between patients with and without changes in diagnosis will be conducted.

#### 2.7.3. Quality assessment

To ensure the quality of the study, several precautions have been taken. Credibility will be strengthened by the prolonged engagement of the main researcher in the field of research on mental health for asylum seekers (75, 76). Although it is practically impossible to execute formal member checking, summary questions will be used during the additional CFI modules to verify that the interviewer has accurately understood the participant’s experience, with the request of correcting any misunderstandings. Peer debriefings take place every day interviews are conducted, to improve the skills of data collectors, gain real-time insights into the data, adapt to unexpected changes and challenges, and increase the accuracy and trustworthiness of the data (75–77). The participant’s caregiver, an independent experienced clinical psychiatrist (variable), an expert psychiatrist supervising the study (SVDA), and the main researcher-psychiatrist (LC) will be present during the multidisciplinary case discussions. This permits a vast moment of data triangulation, evaluating the data from different angles. Methodological triangulation, by utilizing various methods to gather and examine data, will enhance the credibility and trustworthiness of the findings (78).

This protocol paper aims to guarantee transparency and enhance transferability, providing a thorough description of the context, setting, participants, and methodology (79). Collaboration with field workers during the development and execution of this study will guarantee the rootedness in, and relevance for the clinical practice of our findings.

The main researcher (LC) will lead the coding and analyzing process. To maximize the dependability of our study, the general aim is to double-code 20% of the data and to let a supervising team member (SVDA) check and validate the codes and themes (75). Consistency will be assured through regular team discussions, in which codes and interpretations will be considered. If there are any discrepancies in opinion, a consensus will be sought.

The study team members are all clinical psychiatrists with experience in cultural psychiatry. Commitment and idealism drive the research team of this study to act as advocates for the mental health of vulnerable populations such as asylum seekers. This might impact the attitude toward the findings. The COREQ (COnsolidated criteria for REporting Qualitative research) checklist will be used to report on crucial aspects of the study’s methods, context, findings, analysis, and interpretation (80).

## 3. Discussion

To the best of our knowledge, this study will for the first time evaluate the use of the CFI in asylum seekers in particular. More specifically, we will analyze the value of the CFI in the process of a psychiatric assessment, the content added by the CFI to the illness narratives of asylum seekers, and the asylum seekers’ experience of the CFI. This project answers a knowledge gap on the use of the CFI in asylum seekers and will provide knowledge for guiding further implementation.

Research on the CFI among asylum seekers is important because they experience many barriers in mental healthcare (7). It can help address fieldworkers’ challenges in understanding asylum seekers with mental health issues. The CFI can also support healthcare providers who today report difficulties in dealing with asylum seekers in mental healthcare (9) or refer them to non-psychiatric care (6). Furthermore, asylum seekers often face inadequate mental healthcare, and their needs are regularly overlooked by policymakers (13, 81). Unlocking the potential of the CFI for asylum seekers can be of use on all these levels.

### 3.1. Strengths and limitations

This is the first study that uses the CFI combined with a thorough symptomatic psychiatric evaluation, in a population with exclusively asylum seekers. The strengths of this study are the embedding of the project in the stakeholder’s network, its multimethodological approach, and the clinical relevance of the research questions.

Another strength of the study is the methodological stepped approach for the multidisciplinary case discussions, integrating routine psychometric measurement, representative of standard clinical assessment. This will allow a valid comparison between classic and culturally sensitive psychiatric practices. It also allows for the first time to thoroughly describe and analyze the role of the CFI in the diagnostic process. To ensure credibility, interviews and case presentations will follow an identical pattern and be based on the verbatim transcripts of the interview with the participant.

This innovative approach also implies certain limitations. Although the applicate symptomatic questionnaires have been validated in ethnically different populations, it is possible that misunderstanding or restrictive responsiveness has an impact on the validity of the responses. Previous research also revealed that CFI questions about cultural identity and background can lead to perceptions of “alterity” and distance instead of alliance (82). For the first time research on the experience of CFI will focus exclusively on asylum seekers with their specific legal and uncertain social context. A sense of general distrust may influence the experience of the CFI. Assessing the participants’ experience with a mixed-method study design will facilitate understanding and strengthening of the findings. Given that the debriefing interviews take place with the same researcher as the CFI administration, this might induce social desirability bias.

Certified interpreters will be used if the participant is not fluent in Dutch, French, or English. Working with interpreters will allow the participant to express themselves in their native language. While it may result in translation difficulties, it also ensures clinical representativeness.

The involvement of the researchers in participant interviews allows for the potential introduction of personal bias. This aspect is therefore also recognized by the constructivist stance of this research.

## 4. Ethics and dissemination

Oral and written information for patients, translated into 3 national (English, Dutch, and French) and 3 foreign (Dari, Pashtu, and Arabic) languages, will be given, underlining that participation is voluntary and can be withdrawn at any time without negative consequences. It will be clearly expressed to the patients and included in the informed consent that elements discussed during the different interviews, will not interfere with their asylum procedure. Expectations or fear in this sense will therefore be disproved before the interview. Attention will be paid to the easy comprehensibility of the informed consent. Written informed consent will be asked of all the participants. For minors, an agreement of the legal representative will be requested.

Studies on CFI often excluded asylum seekers from participating, given their restricted access to the healthcare system (15, 83, 84). Therefore, the study protocol was tailored in close collaboration with various stakeholders including those responsible for the reception network and care initiatives for asylum seekers. The availability of mental healthcare, the embedding of this project in these care initiatives, and the extensive debriefing with the involved caregivers permit the execution of this research in an ethically responsible way. Participants with a request for help will be referred to the present caregivers. If the researcher has concerns during the interview that the subject or someone close to them may be in immediate danger, they will inform the caregiver to ensure the participants’ safety. The involved caregiver will be available to answer participants’ questions or complaints during their participation.

The protocol and research procedures have been validated after an initial pilot phase.

Main ethical approval for the study was obtained from the Ethical Review Board of the University of Antwerp (BUN B3002022000005) and of the Ethical Committee of the non-profit organization Brothers of Charity (0G054-2022-09). All the involved healthcare institutions and asylum centers gave their written consent for participation. The study will be executed in accordance with the good clinical (GCP) guidelines, the ethical principles outlined in the Declaration of Helsinki, and the general data protection regulation (GDPR).

This research has been designed to create knowledge on the CFI’s content, experience, and added value in working with asylum seekers. The findings will be disseminated via peer-reviewed publications, conference presentations, and lay reports. Feedback moments for caregivers will be organized (e.g., workshops), during which findings will be member checked. Out of this interaction, training materials and recommendations for different policy levels will be formulated. Participation in the public debate will be undertaken to defend the interests and needs of asylum seekers.

## Author contributions

LC is the main researcher of this project and has written the initial draft of this protocol study. SA and BS are involved as supervisors of the research project. MS-O and MB are members of the research project’s scientific board. GB functions as a coordinator of the project within the NGO “Brothers of Charity.” SA reworked the first review of the manuscript. BS, MS-O, MB, and GB reviewed the manuscript’s revised version. All authors contributed to the article and approved the submitted version.

## Funding

This research project has received a grant (no. 2021-J5200810-219976) from the King Baudouin Foundation (Belgium), within the UCB Community Health Fund framework. The initial research project was called “Impact of COVID-19 pandemic on the well-being and health of young asylum seekers and unaccompanied refugee minors with mild and severe psychiatric conditions in Belgium.” The decision to focus on adolescents (15–29 years old) in this study was influenced by the funding criteria set forth by the funder. Publication of this article has been supported by the University Foundation of Belgium (WA-0440).

## Conflict of interest

The authors declare that the research was conducted in the absence of any commercial or financial relationships that could be construed as a potential conflict of interest.

## Publisher’s note

All claims expressed in this article are solely those of the authors and do not necessarily represent those of their affiliated organizations, or those of the publisher, the editors and the reviewers. Any product that may be evaluated in this article, or claim that may be made by its manufacturer, is not guaranteed or endorsed by the publisher.
